# Electronic and optical responses of quasi-one-dimensional phosphorene nanoribbons to strain and electric field

**DOI:** 10.1038/s41598-018-24521-w

**Published:** 2018-04-17

**Authors:** Longlong Zhang, Yuying Hao

**Affiliations:** College of Physics and Optoelectronics, Taiyuan University of Technology, Taiyuan, 030024 China

## Abstract

Electronic and optical responses of zigzag- and armchair-edge quasi-one-dimensional phosphorene nanoribbons (Q1D-PNRs) to strain and external field are comparatively studied based on the tight-binding calculations. The results show that: (i) Zigzag-edge Q1D-PNR has the metallic ground state; applying global strains can not open the gap at the Fermi level but applying the electric field can achieve it; the direct/indirect character of the field-induced gap is determined by the electron-hole symmetry; an electric-field-enhanced optical absorption of low-energy photons is also predicted. (ii) Armchair-edge Q1D-PNR turns out an insulator with the large direct band gap; the inter-plane strain modulates this gap non monotonically while the in-plane one modulates it monotonically; in addition, the gap responses to electric fields also show strong direction dependence, i. e., increasing the inter-plane electric field will monotonically enlarge the gap but the electric field along the width direction modulates the gap non monotonically with three characteristic response regions.

## Introduction

Black phosphorus (BP) is the most stable allotrope of phosphorus and its bulk type was early discovered a century years ago^1^. In recent years, few-layer^2,3^ and single-layer BP^4,5^ were successfully exfoliated from the bulk ones and again inspired peoples’s great enthusiasm to investigate their electronic^6,7^ and optical properties^8^. The single-layer BP is the typical two-dimensional (2D) material consisting of single nonmetal atom, which is similar to graphene and thus usually called phosphorene^3,4^. Unlike graphene, phospherene is a semiconductor with direct band gap and has highly anisotropic character on the electrical conductivity^9^, thermal conductivity^10,11^, and optical response^8^. These excellent characters make phosphorene a promising candidate for application as thin film electronics, infrared optoelectronics and novel devices with anisotropic properties^2,8,9,12–14^.

The gap size of pristine phosphorene is about 1.8 eV and can be modulated mechanically or electronically. One of the widely used approach is the proper utilization of strain^15–17^. For example, it was reported that under the compressive in-plane strain phosphorene would become the indirect-gap insulators^3^, while under the inter-plane compressive strain an insulator-metal (I-M) was expected^15^. On the other hand, phosphorene exhibits excellent response ability to static external electric field. Several experimental/theoretical work suggested BP’s most perspective application as field-effect transistors (FETs)^7,18–20^.

Similar to graphene, phosphorene can be cut or tailored into derived nanostructures. The electronic properties of the phosphorene nanoribbons (PNRs) are strongly dependent on their edge shapes. Depending on the cutting orientation and the way of termination, one can get zigzag-, armchair- and cliff-edge PNRs. Recently, few-nanometer-wide PNRs were successfully derived using a top-down method^21^. Another group further reported the realization of BP atomic chains via electron beam ring inside a transmission electron microscope^22^. These experimental work suggest the perspective to develop BP-based quasi-one-dimensional (Q1D) molecular devices. But by now, the detailed information of Q1D-PNRs’ electronic/optical response to strain/electric-field is still not clear enough, which is however essentially important for determining the Q1D-PNRs’ application perspectives.

**Figure 1:**
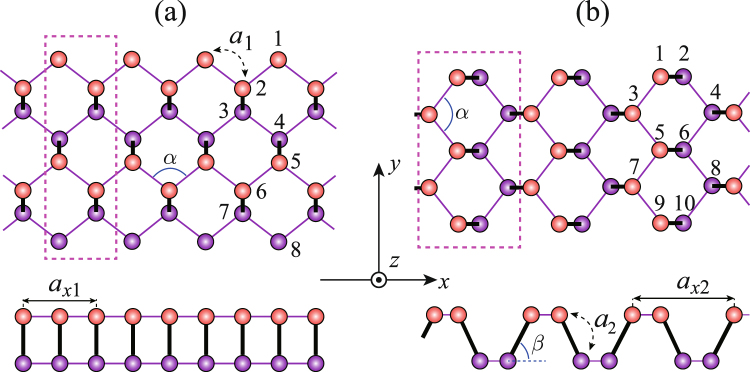
Top and side views of (**a**) 4z-Q1D-PNR and (**b**) 5a-Q1D-PNR. The orange and blue balls represent the upper-plane phosphorus atoms and the lower-plane ones. Unit cells are pointed out in the dashed blanks. Phosphorus atoms on the edges are all H-passivated.

To clarify the above concern, we respectively carry out the tight-binding (TB) calculations^23–26^ on the zigzag and armchair-edge Q1D-PNR, which are shown in Fig. 1. The zigzag-edge Q1D-PNR in Fig. 1(a) is named for 4z-Q1D-PNR since it consists of four zigzag phosphorus chains, while the armchair-edge Q1D-PNR in Fig. 1(b) is named for 5a-Q1D-PNR because its each unit cell consists of five inter-plane P-P couples. For both the two, the electronic/optical responses to strain/electric field are theoretically studied in the rest of this paper.

## TB Model

**Figure 2:**
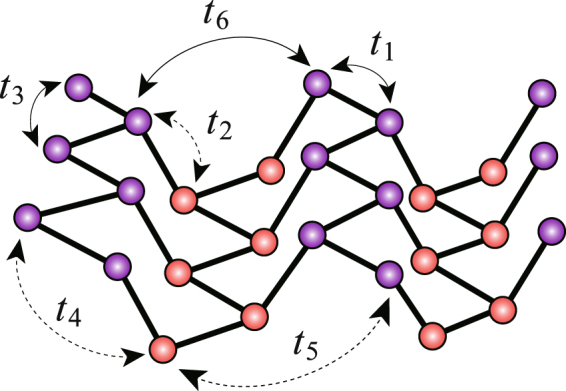
Schematic representation of the hopping integrals involved in the present TB model.

TB model calculations have been proved successful to describe the ground-state electronic/optical properties of BP layers and ribbons by comparing to the *ab initial* results^23–26^. In refs^23,24^. Rudenko *et al*. proposed the TB model for layered BP which consists of 10 in-plane hopping integrals and 4 inter-plane ones. In this work, we simplify that into a simple one with only 6 hopping integrals which are depicted in Fig. 2. With further taking into account the electron-electron (e-e) interactions^27^, the final resulting TB Hamiltonian reads as:

1 $$\begin{array}{rcl} {\mathcal H}  & = & \,-\,\,\sum _{ < i,j > ,s}{t}_{i,j}({c}_{i,s}^{\dagger }{c}_{j,s}+h.\,c.)+U\sum _{i}({c}_{i,\uparrow }^{\dagger }{c}_{i,\uparrow }-\frac{1}{2})({c}_{i,\downarrow }^{\dagger }{c}_{i,\downarrow }-\frac{1}{2})\\  &  & +{V}_{i,j}\sum _{ < i,j > }\sum _{s,s^{\prime} }({c}_{i,s}^{\dagger }{c}_{i,s}-1)({c}_{j,s^{\prime} }^{\dagger }{c}_{j,s^{\prime} }-1)\mathrm{.}\end{array}$$

The summation ${\sum }_{ < i,j > }$ runs over the considered hopping integrals. Operator ${c}_{i}^{\dagger }$ (*c*_*i*_) is to create (annihilate) a *p*_*z*_ orbital electron with spin *s* at site *i*; *t*_*i,j*_ represents the hopping integral between sites *i* and *j*. The value of *t*_*i,j*_ depends on the relative angle and distance between site *i* and *j*^17^. For the fully relaxed puckered structure, *t*_*i,j*_ is determined by reproducing the first-principle calculations. We set the hopping integral between the nearest-neighboring (NN) sites as *t*_1_ = −1.22e*V*. Meanwhile, the other parameters are set for: *t*_2_ = −2.5*t*_1_, *t*_3_ = 0.17*t*_1_, *t*_4_ = 0.01*t*_1_, *t*_5_ = 0.05*t*_1_, *t*_6_ = 0.02*t*_1_^23–26^. Obviously, it can be a reasonable approximation to further set *t*_4_ = *t*_5_ = *t*_6_ = 0 because they are much smaller than *t*_1_, *t*_2_ and *t*_3_. Indeed, we numerically checked that no qualitative difference was induced by this approximation. *U* denotes the on-site e-e interaction while the inter-site ones are modeled in ${V}_{i,j}=U/\kappa \sqrt{1+0.6117{r}_{i,j}^{2}}$, where *κ* = 1.5 reads as a dielectric parameter and *r*_*i,j*_ the distance between the sites *i* and *j*. Referring to Fig. 1, we set the structural parameters as follows: the distance between the in-plane NN sites *a*_1_, and that between the inter-plane NN sites *a*_2_, are equally set as ${a}_{1}={a}_{2}=2.16{\rm{\AA }}$^26^; the angular parameters are set as *β *= 104°, *α* = 98°. E-e interactions are treated by the Hartree-Fock (HF) approximations.

## Results

### 4z-Q1D-PNR

By applying a Fourier transformation, the real-representation Hamiltonian in Eq. (1) is transformed into the block-diagonalized one in the momentum-representation, which reads as ${\mathcal H} ={\sum }_{{k}_{x},s}{c}_{{k}_{x},s}^{\dagger }{\hat{H}}_{{k}_{x},s}{c}_{{k}_{x},s}$. Each block ${\hat{H}}_{{k}_{x},s}({k}_{x}=\frac{2i\pi }{N{a}_{x1}};\,i=0,1,\ldots ,N;\,{a}_{x1}=2{a}_{1}\,\sin \,\frac{\alpha }{2})$ is an Hermitian conjugated matrix of order eight:

2 $${\hat{H}}_{{k}_{x},s}=[\begin{array}{cccccccc}{D}_{\mathrm{1,1}} & {X}_{\mathrm{1,2}} & 0 & 0 & 0 & 0 & 0 & 0\\ {X}_{\mathrm{1,2}}^{\ast } & {D}_{\mathrm{2,2}} & {Y}_{\mathrm{2,3}} & 0 & 0 & 0 & 0 & 0\\ 0 & {Y}_{\mathrm{2,3}}^{\ast } & {D}_{\mathrm{3,3}} & {X}_{\mathrm{3,4}} & 0 & 0 & 0 & 0\\ 0 & 0 & {X}_{\mathrm{3,4}}^{\ast } & {D}_{\mathrm{4,4}} & {Y}_{\mathrm{4,5}} & 0 & 0 & 0\\ 0 & 0 & 0 & {Y}_{\mathrm{4,5}}^{\ast } & {D}_{\mathrm{5,5}} & {X}_{\mathrm{5,6}} & 0 & 0\\ 0 & 0 & 0 & 0 & {X}_{\mathrm{5,6}}^{\ast } & {D}_{\mathrm{6,6}} & {Y}_{\mathrm{6,7}} & 0\\ 0 & 0 & 0 & 0 & 0 & {Y}_{\mathrm{6,7}}^{\ast } & {D}_{\mathrm{7,7}} & {X}_{\mathrm{7,8}}\\ 0 & 0 & 0 & 0 & 0 & 0 & {X}_{\mathrm{7,8}}^{\ast } & {D}_{\mathrm{8,8}}\end{array}]\mathrm{.}$$

The diagonal elements in Eq. (2) read as

3 $${D}_{m,m}=-\,2{t}_{3}\,\cos (2{k}_{x}{a}_{1}\,\sin \,\frac{\alpha }{2})+U({\rho }_{m,-s}-\frac{1}{2})+V\sum _{m^{\prime} }({\rho }_{m^{\prime} }-\mathrm{1)},$$

where ${\rho }_{m^{\prime} }=\frac{1}{N}{\sum }_{{k^{\prime} }_{x},s^{\prime} }\langle {c}_{{k^{\prime} }_{x},m^{\prime} ,s^{\prime} }^{\dagger }{c}_{{k^{\prime} }_{x},m^{\prime} ,s^{\prime} }\rangle$ is the average charge density at site *m*′. The off-diagonal matrix elements include two types, the effective in-plane hopping integrals *X*_*m,n*_ and the inter-plane ones *Y*_*m,n*_. *X*_*m,n*_ are written in

4 $${X}_{m,n}=-\,2{t}_{1}\,\cos ({k}_{x}{a}_{1}\,\sin \,\frac{\alpha }{2})-2V[\cos ({k}_{x}{a}_{1}\,\sin \,\frac{\alpha }{2}){p}_{m,n,s}+\,\sin ({k}_{x}{a}_{1}\,\sin \,\frac{\alpha }{2}){q}_{m,n,s}],$$

where bond orders *p*_*m,n,s*_ and *q*_*m,n,s*_ are derived as

5 $$\begin{array}{c}{p}_{m,n,s}=\frac{1}{N}\sum _{{k^{\prime} }_{x}}\langle {c}_{{k^{\prime} }_{x},n,s}^{\dagger }{c}_{k^{\prime} ,m,s}\rangle \cos ({k^{\prime} }_{x}{a}_{1}\,\sin \,\frac{\alpha }{2}),\\ {q}_{m,n,s}=\frac{1}{N}\sum _{{k^{\prime} }_{x}}\langle {c}_{k^{\prime} ,n,s}^{\dagger }{c}_{{k^{\prime} }_{x},m,s}\rangle \sin ({k^{\prime} }_{x}{a}_{1}\,\sin \,\frac{\alpha }{2}),\end{array}$$

with (*m*, *n*) denoting the site couples of (1, 2), (3, 4), (5, 6) and (7, 8). *Y*_*m,n*_ are derived as

6 $${Y}_{m,n}=-\,{t}_{2}-V\sum _{{k^{\prime} }_{x}}\langle {c}_{{k^{\prime} }_{x},n,s}^{\dagger }{c}_{{k^{\prime} }_{x},m,s}\rangle ,$$

where (*m*, *n*) represents the site couples of (2, 3), (4, 5) and (6, 7). We calculate the local density of state (LDOS) at site *i* in the momentum representation by

7 $${\rho }_{i}(E)=\sum _{{k}_{x},s}\sum _{\lambda }\delta (E-{E}_{\lambda }){{\rm{\Psi }}}_{\lambda ,s}^{\ast }({k}_{x},i){{\rm{\Psi }}}_{\lambda ,s}({k}_{x},i),$$

where *E*_*λ*_ is the energy of the *λ*-th HF level and Ψ_*λ,s*_(*k*_*x*_,*i*) denotes the wavefunction of wavevector-*k*_*x*_, spin-*s* and energy-*E*_*λ*_.

**Figure 3:**
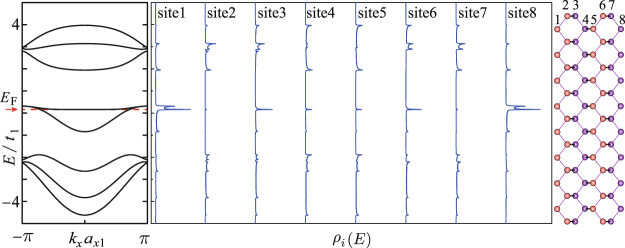
Dispersion relations *E*(*k*_*x*_) and the local density of state *ρ*_*i*_(*E*) of 4z-Q1D-PNR. The Fermi level *E*_F_ = 0.24*t*_1_ is illustrated by the horizontal dashed red line.

Figure 3 presents the ground-state dispersion relations and LDOS of 4z-Q1D-PNR. As shown, the molecular orbital of the *p*_*z*_ electrons are unfolded into eight band branches, among which the ones above (below) the Fermi level (*E*_F_ = 0.24*t*_1_) are numbered as *J*_c_(*J*_v_) = 1.2, $\cdots$ . The electronic state turns out metallic meanwhile the adhesively paired bands are formed around the Fermi level. This band structure is characteristic for zigzag-edge PNRs and have been repeatedly revealed^25,28,29^. By observing the LDOS spectra, one can learn that the electronic state corresponding to the adhesively paired bands are mainly distributed along the ribbon edges, so to say, they are the so-called edge states. The point is that for a zigzag-edge Q1D-PNR, even its ribbon width is extremely narrow, the edge state should still be formed. In the following, we will reveal that the key factor to determine the formation of the paired edge state is the magnitude of the inter-plane hopping integrals.

Then we proceed to discuss the electronic/optical responses of 4z-Q1D-PNR to strains. The optical response is examined by calculating the polarized optical conductivities whose real part is derived as

8 $${\sigma }_{x(y)}=\frac{\pi }{\omega }\sum _{l}{|\langle {E}_{l}|{{\mathscr{J}}}_{x(y)}|{E}_{0}\rangle |}^{2}\delta ({E}_{l}-{E}_{0}-\hslash \omega )\mathrm{.}$$

operator ${{\mathscr{J}}}_{x(y)}$ denotes the current operator with the polarization direction along *x*(*y*) direction. $|{E}_{l}\rangle ={c}_{{k}_{x},{J}_{{\rm{c}}},s}^{\dagger }{c}_{{k}_{x},{J}_{{\rm{v}}},s}|{E}_{0}\rangle$ denotes the *l*-th excited state with the exciting energy ${E}_{l}={E}_{{k}_{x},{J}_{{\rm{c}}}}-{E}_{{k}_{x},{J}_{{\rm{v}}}}$. $|{E}_{0}\rangle ={\prod }_{{k}_{x}}{\prod }_{{J}_{{\rm{v}}}}{c}_{{k}_{x},{J}_{{\rm{v}}},s}^{\dagger }\mathrm{|0}\rangle$ is the HF ground state and |0〉 is the true electron vaccum. Following the way of defining the current operator in ref.^30^, *J*_*x*_ and *J*_*y*_ are respectively derived as

9 $$\begin{array}{rcl}{{\mathscr{J}}}_{x} & = & \frac{-2ie{a}_{1}{t}_{1}}{\hslash }\,\sin \,\frac{\alpha }{2}\sum _{{k}_{x},s}\sin ({k}_{x}{a}_{1}\,\sin \,\frac{\alpha }{2})({c}_{{k}_{x}\mathrm{,1,}s}^{\dagger }{c}_{{k}_{x}\mathrm{,2,}s}\\  &  & +{\,c}_{{k}_{x}\mathrm{,4,}s}^{\dagger }{c}_{{k}_{x}\mathrm{,3,}s}+{c}_{{k}_{x}\mathrm{,5,}s}^{\dagger }{c}_{{k}_{x}\mathrm{,6,}s}+{c}_{{k}_{x}\mathrm{,8,}s}^{\dagger }{c}_{{k}_{x}\mathrm{,7,}s}-h.\,c.\,)\\  &  & \,-\frac{4ie{a}_{1}{t}_{3}}{\hslash }\,\sin \,\frac{\alpha }{2}\sum _{{k}_{x},s}\sum _{m\mathrm{=1}}^{8}\sin (2{k}_{x}{a}_{1}\,\sin \,\frac{\alpha }{2}){c}_{{k}_{x},m,s}^{\dagger }{c}_{{k}_{x},m,s},\end{array}$$

10 $$\begin{array}{rcl}{{\mathscr{J}}}_{y} & = & \frac{-2ie{a}_{1}{t}_{1}}{\hslash }\,\cos \,\frac{\alpha }{2}{\sum }_{{k}_{x},s}\cos ({k}_{x}{a}_{1}\,\sin \,\frac{\alpha }{2})({c}_{{k}_{x}\mathrm{,1,}s}^{\dagger }{c}_{{k}_{x}\mathrm{,2,}s}\\  &  & +{c}_{{k}_{x}\mathrm{,3,}s}^{\dagger }{c}_{{k}_{x}\mathrm{,4,}s}+{c}_{{k}_{x}\mathrm{,5,}s}^{\dagger }{c}_{{k}_{x}\mathrm{,6,}s}+{\,c}_{{k}_{x}\mathrm{,7,}s}^{\dagger }{c}_{{k}_{x}\mathrm{,8,}s}-h\mathrm{.}c\mathrm{.})\\  &  & \,-\frac{ie{a}_{2}{t}_{2}}{\hslash }\,\cos \,\beta \sum _{{k}_{x},s}({c}_{{k}_{x}\mathrm{,2,}s}^{\dagger }{c}_{{k}_{x}\mathrm{,3,}s}+{c}_{{k}_{x}\mathrm{,4,}s}^{\dagger }{c}_{{k}_{x}\mathrm{,5,}s}+{c}_{{k}_{x}\mathrm{,6,}s}^{\dagger }{c}_{{k}_{x}\mathrm{,7,}s}-h\mathrm{.}c\mathrm{.})\mathrm{.}\end{array}$$

**Figure 4:**
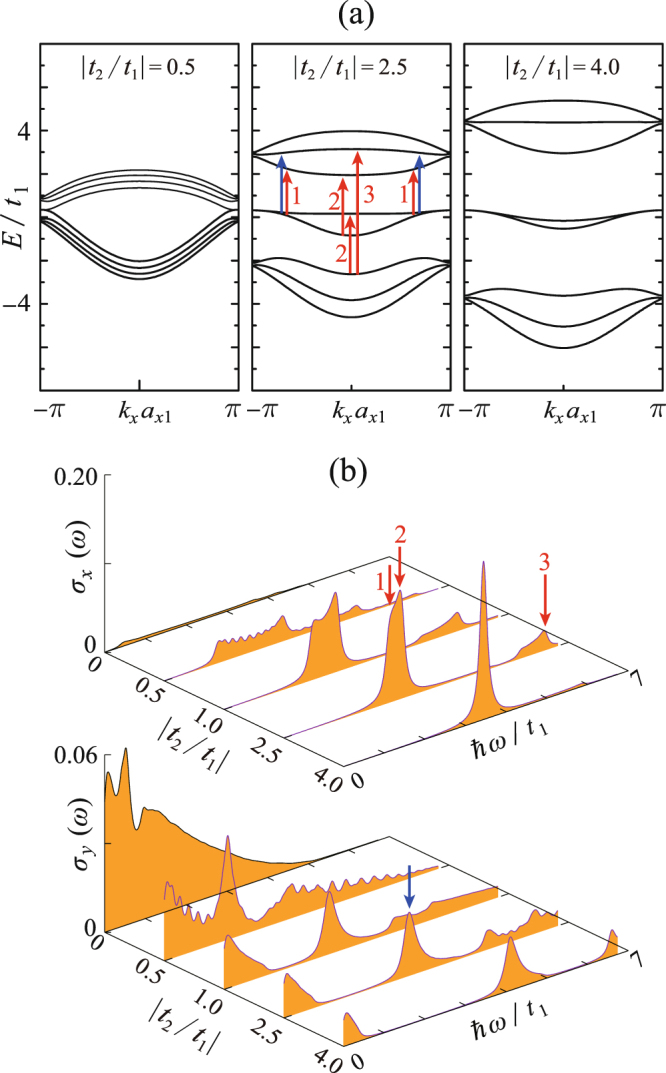
(**a**) Dispersion relations of 4z-Q1D-PNR with varying |*t*_2_/*t*_1_|; (**b**) the *x*-polarized optical conductivity spectra *σ*_*x*_ and the *y*-polarized ones *σ*_*y*_ as functions of |*t*_2_/*t*_1_|. The interband transitions contributing to the absorptions peaks are pointed out by the red (for *x*-polarization) and blue (for *y*-polarization) arrows.

Because PNRs demonstrate superior flexibility and can withstand high tensile strain up to 40%^31^, in this work, we adjust *t*_2_ and *t*_3_ in the large scales. Figure 4 presents 4z-Q1D-PNR’s electronic/optical responses to the inter-plane strain, which is simulated by adjusting the magnitude of |*t*_2_/*t*_1_|. Dispersion relations for three typical valuse |*t*_2_/*t*_1_| = 0.5, 2.5 and 4.0 (respectively correspond to the tensile, none, and compressive inter-plane strain) are demonstrated. Comparing them three, it is found that the formation of the adhesively paired bands strongly depends on the magnitude of |*t*_2_/*t*_1_|. A larger |*t*_2_/*t*_1_| tends to separate the paired bands from the bulked ones and thus eventually leads to the edge state. On the other hand, the optical response to the inter-plane strain is significant [see Fig. 4(b)]. When |*t*_2_/*t*_1_| = 0, 4z-Q1D-PNR can not absorb the *x*-direction polarized photons, but it is easy to absorb the *y*-direction polarized photons at the low-energy regime. With increasing |*t*_2_/*t*_1_|, the absorption of *x*-polarized photons becomes allowed and the corresponding spectrum becomes narrower and higher. When |*t*_2_/*t*_1_| = 4.0, namely, under the strong compressive strain, the optical absorption has already become sharply peaked.

Obviously, the optical selection rules between the *x* and *y* direction is quite distinct. This can be explained by a similar mechanism which we revealed in the previous work^30^. 4z-Q1D-PNR exhibits the *C*_2*x*_ symmetry and there are the relations *C*_2*x*_·${{\mathscr{J}}}_{x}={{\mathscr{J}}}_{x}\,{\rm{and}}\,{C}_{2x}\cdot {{\mathscr{J}}}_{y}=-\,{{\mathscr{J}}}_{y}$. On the other hand, the spatial wave functions of the bands alternatively exhibit symmetrical and antisymmetrical parity. In order to ensure the inner product among the initial and the final state on *J*_*x*(*y*)_ is nonzero, the transitions between the same-parity bands should be only allowed for the *x*-polarized photons but forbidden for the *y*-polarized ones and vice versa. The paired bands near the Fermi level are of opposite parity and therefore we only observe the Drude-like absorptions for the *y*-direction polarized photons.

**Figure 5:**
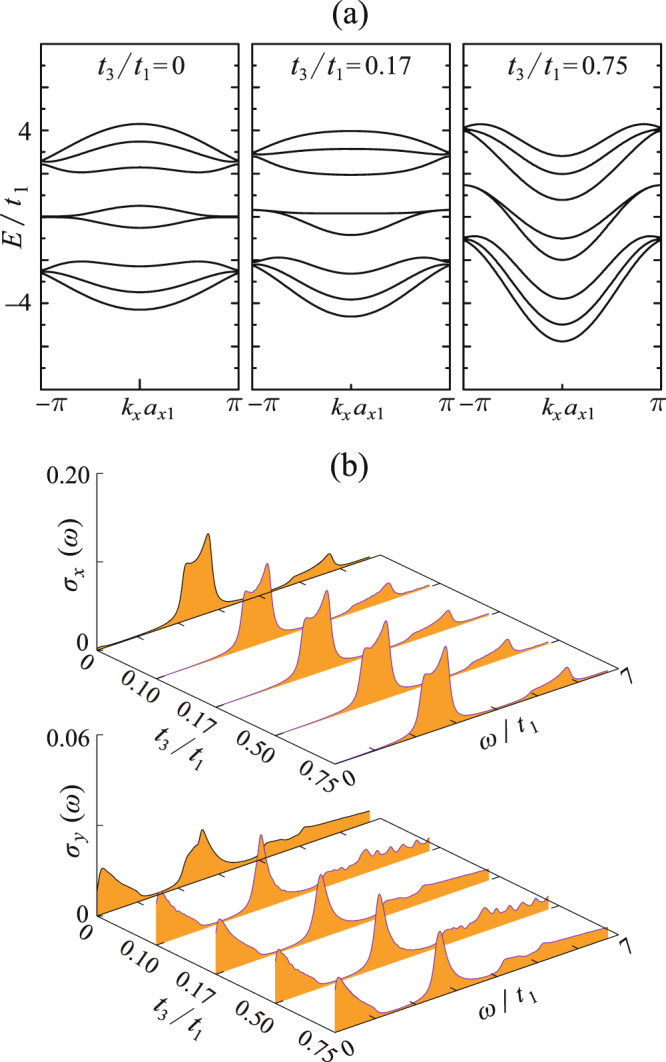
Same as Fig. 4, but for the case of varying *t*_3_/*t*_1_.

Figure 5 shows the in-plane strain’s effects on the electronic/optical properties of 4z-Q1D-PNR. The in-plane strain will significantly change the angle *α*, therefore it primely influences the magnitude of *t*_3_/*t*_1_. In this work, we examine *t*_3_/*t*_1_ in the range from 0 to 0.75 with the nominal value being *t*_3_/*t*_1_ = 0.17. For the limiting case *t*_3_/*t*_1_ = 0, the valence and conduction bands show exact electron-hole (e-h) symmetry, indicating the magnitude of *t*_3_/*t*_1_ is the key factor to determine the e-h asymmetry of PNRs. This conclusion agrees with the experience in graphene nanoribbons that a finite next-nearest-neighbor (NNN) hopping integral will break the e-h asymmetry^32^. Furthermore, with increasing *t*_3_/*t*_1_, the bands tend to show the strong dispersive character, implying the effective mass of electrons/holes will be significantly modulated by the in-plane strains. On the other hand, for the optical properties [Fig. 5(b)], it seems that although the e-h asymmetry is enhanced by increasing *t*_3_/*t*_1_, the optical conductivity spectra are almost not affected. We have checked that the energy difference between the band branches at all the *k* points are almost unchanged for any *t*_3_/*t*_1_, meanwhile the optical selection rules are also maintained against varying *t*_3_/*t*_1_.

**Figure 6:**
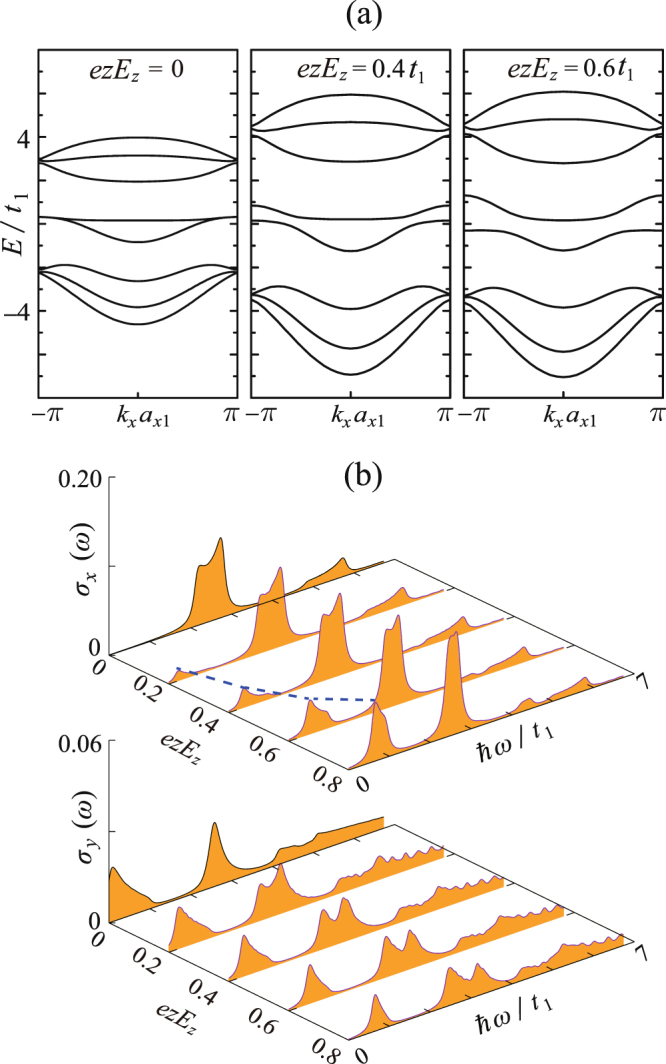
Same as Fig. 4, but for the case of varying the *z*-direction electric fields. The growth of the low-lying *x*-polarized absorption peak with increasing *E*_*z*_ is illustrated by the blue dashed curve in (**b**).

**Figure 7:**
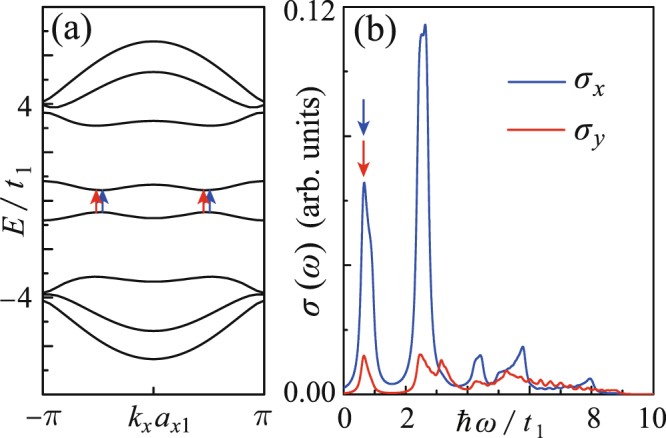
(**a**) When *t*_3_/*t*_1_ = 0 and *eE*_z_z = 0.6*t*_1_, the electronic band structure shows the e-h symmetry and thus the electric-field-induced band gap is direct. (**b**) The electric-field induced absorptions of the *x*- and *y*-polarized photons with energy resonating to the gap.

We notice that for both applying the in-plane and inter-plane strains, the adhesively paired bands do not split to form a gap. This is due to a global strain itself can not break the original symmetry. But applying an external electric field can achieve this goal. For example, Ezawa theoretically revealed the metal-insulator (M-I) transitions induced by applying the *y*-direction electric field^25^. In this paper, we demonstrate that same effects can be also achieved by applying the *z*-direction electric field. If only considering the unscreened electric field, the field induced electronic potential difference between the two planes can be simply estimated as *eE*_*z*_z, where *E*_*z*_ represents the field strength and *z* = *a*_2_cos*β* is the vertical distance between the two planes. The results show that increasing the *z*-direction electric field will indeed trigger the M-I transitions [see Fig. 6(a)] with the transition threshold about *eE*_*z*_*z* ≈ 0.2*t*_1_. The mechanism of the M-I transition is as follows: *z*-direction electric field causes the on-site energy difference between the two planes, which induces the charge redistribution and therefore breaks the original symmetrical properties of bands *J*_*c*_ = 1 and *J*_*v*_ = 1. These two bands will repel each other due to the so-called field-induced anticrossing effect^33–35^ meanwhile the on-site energy difference provides the splitting energy to separate them. It is worth noting that the electric-field-induced gap here is indirect. We find that whether this gap is direct or indirect is dependent on that whether the e-h symmetry of the original zPNR is breaking or not. For example when *t*_3_/*t*_1_ = 0, the e-h symmetry is completely satisfied. In this case, applying the electric field will result in the directly gapped state (Fig. 7). Besides, we also find an interesting phenomenon of the optical response to electric field: the absorption of the low-lying (resonant to the gap size) *x*-polarized photons becomes allowed due to the existence of the electric field. Further increasing the field strength will continuously enhance this absorption. Meanwhile, as the gap is enlarged with increasing the field strength, the low-lying absorption peak behaves a blue shift [see Fig. 6(b)].

We briefly discuss the Coulomb interaction’s effects on the electronic structure of 4z-Q1D-PNR. It is found that increasing Coulomb interaction results in the long-range charge-order-waves (CDWs) along two zigzag edges, while charges almost keep averagely distributed over the inner phosphorus atoms. Even though CDWs are formed along the edge, it fails to open a gap at the Fermi level. Coulomb interaction’s effect is only to enlarge the gap between bands *J*_*c*_ = 2 and 3 and that between *J*_*v*_ = 2 and 3, but has weak influences on that between *J*_*v*_ = 1 and *J*_*c*_ = 1.

### 5a-Q1D-PNR

Generally, armchair-edge PNRs are insulators and thus their electronic properties are qualitatively distinct to those of zigzag-edge ones. In this section, we theoretically investigate 5a-Q1D-PNR’s electronic/optical responses to strain and electric field.

The *k*_*x*_-block Hamiltonian of 5a-Q1D-PNR in the momentum-representation is:

11 $${\hat{H}}_{{k}_{x},s}=[\begin{array}{cccccccccc}{D}_{\mathrm{1,1}} & {Y}_{\mathrm{1,2}} & {X}_{\mathrm{1,3}} & 0 & {Q}_{\mathrm{1,5}} & 0 & 0 & 0 & 0 & 0\\ {Y}_{\mathrm{1,2}}^{\ast } & {D}_{\mathrm{2,2}} & 0 & {X}_{\mathrm{2,4}} & 0 & {Q}_{\mathrm{2,6}} & 0 & 0 & 0 & 0\\ {X}_{\mathrm{1,3}}^{\ast } & 0 & {D}_{\mathrm{3,3}} & {Y}_{\mathrm{3,4}} & {X}_{\mathrm{3,5}} & 0 & {Q}_{\mathrm{3,7}} & 0 & 0 & 0\\ 0 & {X}_{\mathrm{2,4}}^{\ast } & {Y}_{\mathrm{3,4}}^{\ast } & {D}_{\mathrm{4,4}} & 0 & {X}_{\mathrm{4,6}} & 0 & {Q}_{\mathrm{4,8}} & 0 & 0\\ {Q}_{\mathrm{1,5}}^{\ast } & 0 & {X}_{\mathrm{3,5}}^{\ast } & 0 & {D}_{\mathrm{5,5}} & {Y}_{\mathrm{5,6}} & {X}_{\mathrm{5,7}} & 0 & {Q}_{\mathrm{5,9}} & 0\\ 0 & {Q}_{\mathrm{2,6}}^{\ast } & 0 & {X}_{\mathrm{4,6}}^{\ast } & {Y}_{\mathrm{5,6}}^{\ast } & {D}_{\mathrm{6,6}} & 0 & {X}_{\mathrm{6,8}} & 0 & {Q}_{\mathrm{6,10}}\\ 0 & 0 & {Q}_{\mathrm{3,7}}^{\ast } & 0 & {X}_{\mathrm{5,7}}^{\ast } & 0 & {D}_{\mathrm{7,7}} & {Y}_{\mathrm{7,8}} & {X}_{\mathrm{7,9}} & 0\\ 0 & 0 & 0 & {Q}_{\mathrm{4,8}}^{\ast } & 0 & {X}_{\mathrm{6,8}}^{\ast } & {Y}_{\mathrm{7,8}}^{\ast } & {D}_{\mathrm{8,8}} & 0 & {X}_{\mathrm{8,10}}\\ 0 & 0 & 0 & 0 & {Q}_{\mathrm{5,9}}^{\ast } & 0 & {X}_{\mathrm{7,9}}^{\ast } & 0 & {D}_{\mathrm{9,9}} & {Y}_{\mathrm{9,10}}\\ 0 & 0 & 0 & 0 & 0 & {Q}_{\mathrm{6,10}}^{\ast } & 0 & {X}_{\mathrm{8,10}}^{\ast } & {Y}_{\mathrm{9,10}}^{\ast } & {D}_{\mathrm{10,10}},\end{array}]$$

where ${k}_{x}=\frac{2j\pi }{N{a}_{x2}}(j=\mathrm{0,1,}\,\mathrm{...,}\,N)$ and ${a}_{x2}=2{a}_{1}\,\cos \,\frac{\alpha }{2}+2{a}_{2}\,\cos \,\beta$ [see Fig. 1(b)]. The diagonal elements in the above matrix read as

12 $${D}_{m,m}=U({\rho }_{m,-s}-\frac{1}{2})+V\sum _{m^{\prime} }({\rho }_{m^{\prime} }-\mathrm{1)}\mathrm{.}$$

The off-diagonal matrix elements corresponding to the in-plane NN effective-hopping-integrals are defined as

13 $${X}_{m,n}=-\,{t}_{1}\exp [-i{k}_{x}{a}_{1}{\rm{c}}os\frac{\alpha }{2}]-V\exp [-i{k}_{x}{a}_{1}\,\cos \,\frac{\alpha }{2}]{p}_{m,n,s},$$

where

14 $${p}_{m,n,s}=\frac{1}{N}\sum _{{k^{\prime} }_{x}}\langle {c}_{{k^{\prime} }_{x},n,s}^{\dagger }{c}_{{k^{\prime} }_{x},m,s}\rangle \exp (i{k^{\prime} }_{x}{a}_{1}\,\cos \,\frac{\alpha }{2}),$$

and (*m*, *n*) represents the site-couples of (1, 3), (5, 3), (5, 7), (9, 7), (4, 2), (4, 6), (8, 6) and (8, 10) $({X}_{m,n}={X}_{n,m}^{\ast })$. The in-plane NNN effective hopping integrals are defined as

15 $${Q}_{m,n}=-\,{t}_{3}-V{q}_{m,n,s},$$

where

16 $${q}_{m,n,s}=\frac{1}{N}\sum _{{k^{\prime} }_{x}}\langle {c}_{{k^{\prime} }_{x},n,s}^{\dagger }{c}_{{k^{\prime} }_{x},m,s}\rangle ,$$

where (*m*, *n*) represents the site-couples of (1, 5), (2, 6), (3, 7), (4, 8), (5, 9) and (6, 10). The inter-plane effective hopping integrals are defined as

17 $${Y}_{m,n}=-\,{t}_{2}\,\exp \,(\,-\,i{k}_{x}{a}_{2}\,\cos \,\beta )-V\,\exp \,(\,-\,i{k}_{x}{a}_{2}\,\cos \,\beta ){y}_{m,n,s},$$

where

18 $${y}_{m,n,s}=\frac{1}{N}\sum _{{k^{\prime} }_{x}}\langle {c}_{{k^{\prime} }_{x},n,s}^{\dagger }{c}_{k^{\prime} ,m,s}\rangle \exp (i{k^{\prime} }_{x}{a}_{2}\,\cos \,\beta )\mathrm{.}$$

and (*m*, *n*) = (2, 1), (3, 4), (6, 5), (7, 8) and (10, 9).

**Figure 8:**
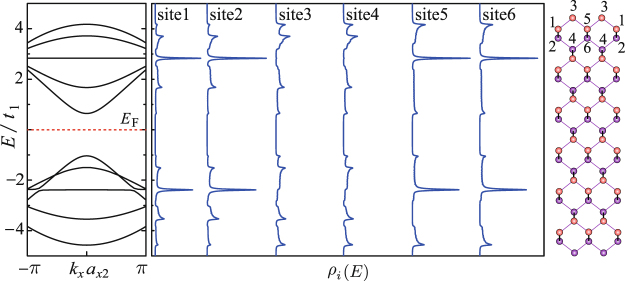
Same as Fig. 3, but for the case of 5a-Q1D-PNR. The Fermi level sits at *E*_F_ = 0.

Figure 8 presents the dispersion relations and LDOS of 5a-Q1D-PNR. As shown, 5a-Q1D-PNR has the direct band gap at *k* = 0. The gap size is about Δ_g_ = 1.7*t*_1_ ≈ 2.1eV. The scaling rule of armchair-edge PNRs’s band-gap with changing the ribbon width has been previously revealed^26,36^. The gap is monotonically reduced with increasing the ribbon width by Δ_g_ ~1/*d*^2^ (*d* the ribbon width). It seems that the band-gap scaling rule of aPNRs does not follow the so-called 3*n*-rule which was found in aGNRs (ribbons with width 3*n* + 2 are nearly metallic)^37,38^. In addition, two flat bands are formed in the 5a-Q1D-PNR’s band structure. Notice that the flat bands are not formed in the gap region but embedded in the bulked valance and conduction bands, therefore, they do not significantly contribute to additional effects on the electronic properties unless it is doped at a proper concentration. This band structure somehow seems similar to that of polyphenanthrene (PPN)^39^, which is the narrowest armchair-edge graphene nanoribbon. There was a famous story that PPN turned out a BCS-type superconductivity with the Curie temperature about 10 K by doping alkali^40^. We notice that a DFT calculation has predicted that similar superconductivity mechanism seems also realizable in aPNRs when it was doped by electrons^41^.

We proceed to study 5a-Q1D-PNR’s electronic/optical responses to strain and electric field. The real part of optical conductivities are calculated using Eq. (8), where the current operators along the *x* and *y* directions are now respectively defined as:

19 $$\begin{array}{rcl}{{\mathscr{J}}}_{x} & = & -\frac{ie{t}_{1}}{\hslash }{a}_{1}\,\cos \,\frac{\alpha }{2}\sum _{{k}_{x},s}\sum _{m,n}[\exp (-i{k}_{x}{a}_{1}\,\cos \,\frac{\alpha }{2}){c}_{{k}_{x},m,s}^{\dagger }{c}_{{k}_{x},n,s}-h.\,c\mathrm{.}]\\  &  & -\frac{ie{t}_{2}}{\hslash }{a}_{2}\,\cos \,\beta \sum _{{k}_{x},s}\sum _{p,q}[\exp (\,-\,i{k}_{x}{a}_{2}\,\cos \,\beta ){c}_{{k}_{x},p,s}^{\dagger }{c}_{{k}_{x},q,s}-h.\,c\mathrm{.}],\end{array}$$

with (*m*, *n*) denoting the site-couples (1, 3), (4, 2), (5, 3), (4, 6), (5, 7), (8, 6), (9, 7) and (8, 10); (*p*, *q*) for the site-couples of (2, 1), (6, 5), (10, 9), (3, 4) and (7, 8);

20 $$\begin{array}{rcl}{{\mathscr{J}}}_{y} & = & -\frac{ie{t}_{1}}{\hslash }{a}_{1}\,\sin \,\frac{\alpha }{2}\sum _{{k}_{x},s}\sum _{m,n}[\exp (-i{k}_{x}{a}_{1}\,\cos \,\frac{\alpha }{2}){c}_{{k}_{x},m,s}^{\dagger }{c}_{{k}_{x},n,s}-h.\,c\mathrm{.}]\\  &  & -\frac{ie{t}_{1}}{\hslash }{a}_{1}\,\sin \,\frac{\alpha }{2}\sum _{{k}_{x},s}\sum _{p,q}[\exp (i{k}_{x}{a}_{1}{\rm{c}}os\frac{\alpha }{2}){c}_{{k}_{x},p,s}^{\dagger }{c}_{{k}_{x},q,s}-h.\,c\mathrm{.}]\\  &  & -\frac{2ie{t}_{3}}{\hslash }{a}_{1}\,\sin \,\frac{\alpha }{2}\sum _{{k}_{x},s}\sum _{j,l}[{c}_{{k}_{x},j,s}^{\dagger }{c}_{{k}_{x},l,s}-h\mathrm{.}c\mathrm{.}],\end{array}$$

with (*m*, *n*) representing for the site-couples of (1, 3), (4, 6), (5, 6) and (8, 10); (*p*, *q*) for (3, 5), (2, 4), (7, 9) and (6, 8); (*j*, *l*) for (1, 5), (2, 6), (5, 9), (6, 10), (3, 7) and (4, 8).

**Figure 9:**
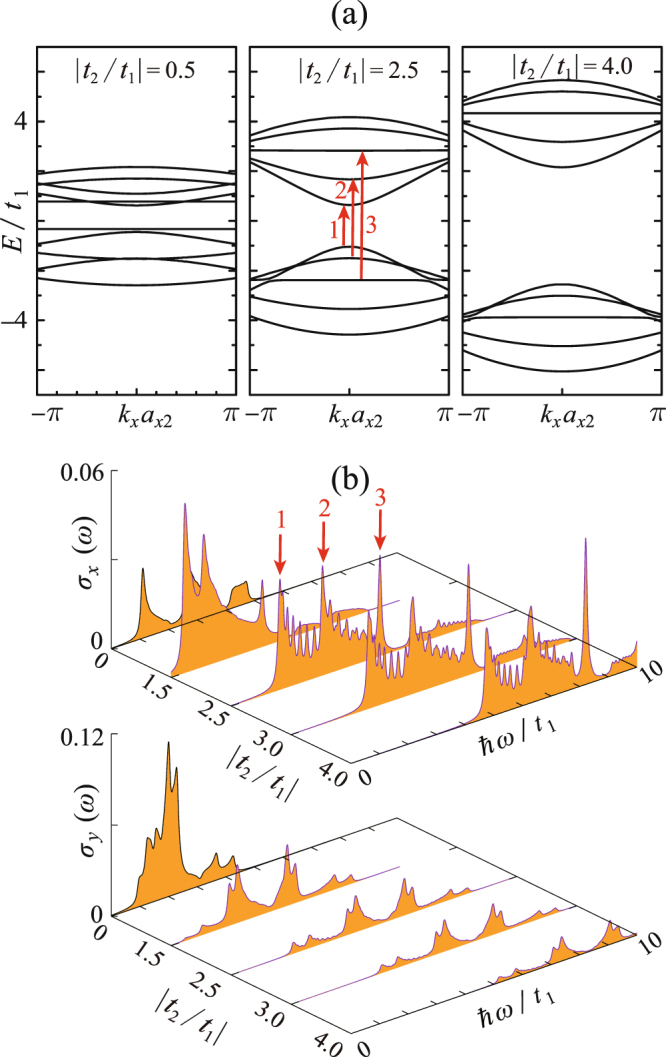
Same as Fig. 4, but for the case of 5a-Q1D-PNR. The interband transitions contributing to the main absorption peaks are pointed out by red arrows.

Figure 9 demonstrates the electronic/optical responses to the inter-plane strain. Fundamentally, we conclude that the tensile strains (e. g. |*t*_2_/*t*_1_| = 0.5) tend to reduce the gap and the band width, while the compressive ones (e. g. |*t*_2_/*t*_1_| = 2.5) tend to enlarge them. Such a trend can be read out from the optical conductivity spectra *σ*_*x*_ and *σ*_*y*_, where the absorption peaks show significant blue shift with increasing |*t*_2_/*t*_1_|. Furthermore, we find that the positions of the two flat bands in the band structure can be modulated by adjusting |*t*_2_/*t*_1_|. For example, when |*t*_2_/*t*_1_| = 0.5, the flat bands almost coincide to Fermi level. In such case, the flat bands may induce some novel phenomena caused from the Van Hove singularity near the Fermi level, e. g. ferromagnetism^42^, fractional Hall effect^43^, and superconductivity^44^. So to say, we predict that beside the way of electronic doping, a tensile strain may also possibly induce a superconductivity in armchair-edge Q1D-PNR.

On the other hand, by comparing the *x*-polarized optical conductivity spectra in Figs 4(b) and 9(b), it can be concluded that the selection rule of PNRs shows strong edge-dependence. This phenomenon can be understood by referring to the scenario in GNRs^45–47^. Lin *et al*. addressed that the selection rule of zGNRs satisfied Δ*J* = |*J*_*c*_−J_v_| = *odd* while the armchair-edge ones were governed by the rule of Δ*J* = 0^33,45,47^. By checking the dipole moment of interband transitions of 4z-Q1D-PNR and 5a-Q1D-PNR contributing to their main absorption peaks [red arrows in Figs 4(b) and 9(b)], we find the edge-dependent selection rules revealed by Lin also work for PNRs.

**Figure 10:**
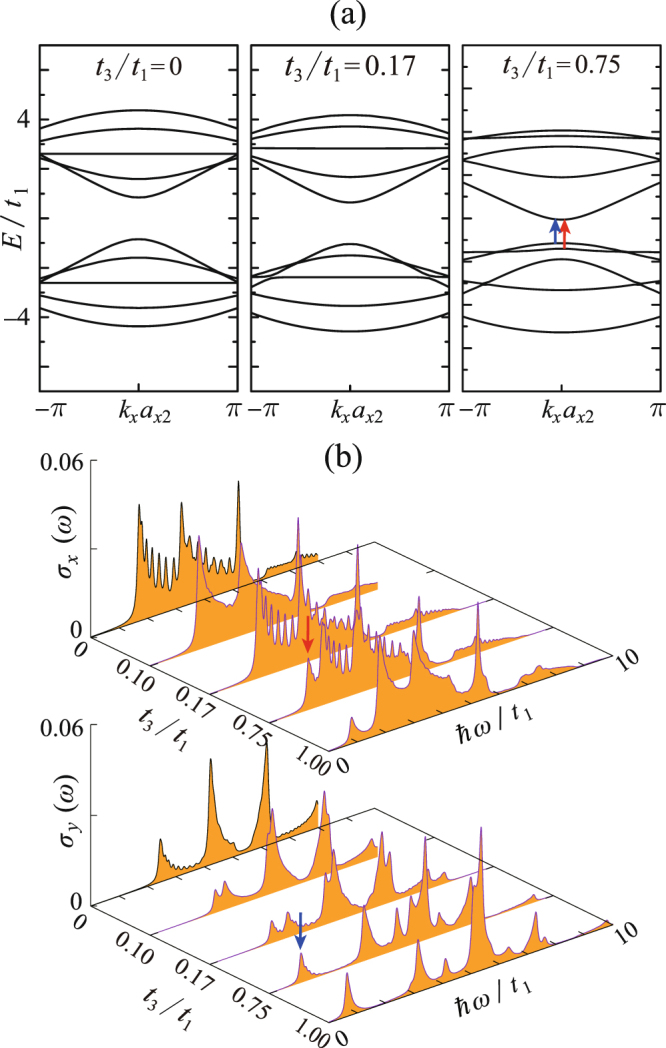
Same as Fig. 5, but for the case of 5a-Q1D-PNR. The lowest-lying absorption peaks and the corresponding interband transitions are illustrated by red (*x*-polarization) and blue (*y*-polarization) arrows.

Figure 10 demonstrates the effects of adjusting *t*_3_/*t*_1_ in 5a-Q1D-PNR. Same to 4z-Q1D-PNR, a finite *t*_3_/*t*_1_ will lead to the e-h asymmetry. In addition, the band gap is significantly decreased with increasing *t*_3_/*t*_1_. Unlike the case of varying |*t*_2_/*t*_1_|, increasing *t*_3_/*t*_1_ will parallelly move the two flat bands in one direction. Meanwhile, the *J*_*c*_ = 1 band becomes more dispersive while the *J*   ^v^ = 1 band becomes more flat with increasing *t*_3_/*t*_1_. These bands’ transitions can be identified from the optical conductivity spectra shown in Fig. 10(b). Increasing *t*_3_/*t*_1_ until the low-energy flat band approaches to the *J*^v^ = 1 band, one satellite absorption peak is separated from the continuum spectra of *σ*_*x*_. We have checked that this satellite peak corresponds to the transitions from the flat band to the *J*_*c*_ = 1 band. On the other hand, because the band gap is continuously decreased with increasing *t*_3_/*t*_1_, one can observe a resultant red shift of the lowest-energy absorption peak in the *σ*_*y*_ spectra.

**Figure 11:**
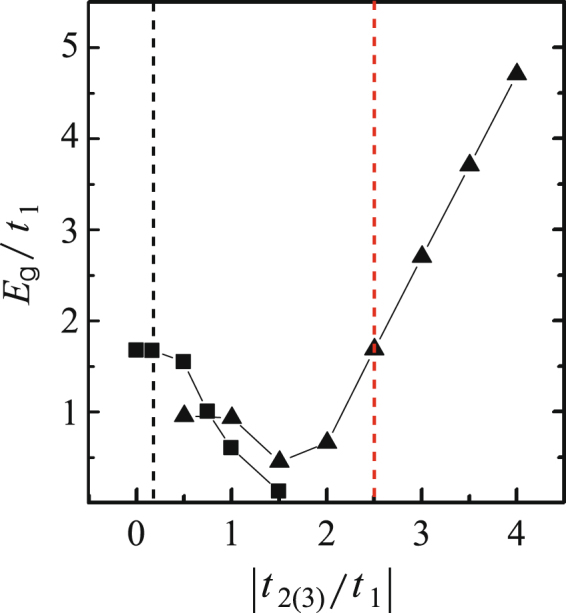
Gap values *E*_*g*_ of 5a-Q1D-PNR as functions of |*t*_2_/*t*_1_| (triangle labelled line) and (*t*_3_/*t*_1_) (square labelled line). The nominal parameters are |*t*_2_/*t*_1_| = 2.5 and *t*_3_/*t*_1_ = 0.17.

The gap size as functions of |*t*_2_/*t*_1_| and *t*_3_/*t*_1_ are summarized in Fig. 11. As shown, adjusting |*t*_2_/*t*_1_| will modulate the gap non monotonically. As the nominal value is |*t*_2_/*t*_1_| = 2.5, fundamentally, we say that a compressive inter-plane strain tends to enlarge the gap while a tensile inter-plane strain tends to reduce the gap. The smallest gap reaches about 0.5*t*_1_ at |*t*_2_/*t*_1_| = 1.5. On the other hand, for adjusting *t*_3_/*t*_1_, it is found that increasing *t*_3_/*t*_1_ leads to the monotonic reduction of the band gap. The gap almost closes when *t*_3_/*t*_1_ = 1.5*t*_1_. In a word, the electronic response of 5a-Q1D-PNR to strains shows strong direction dependence.

**Figure 12:**
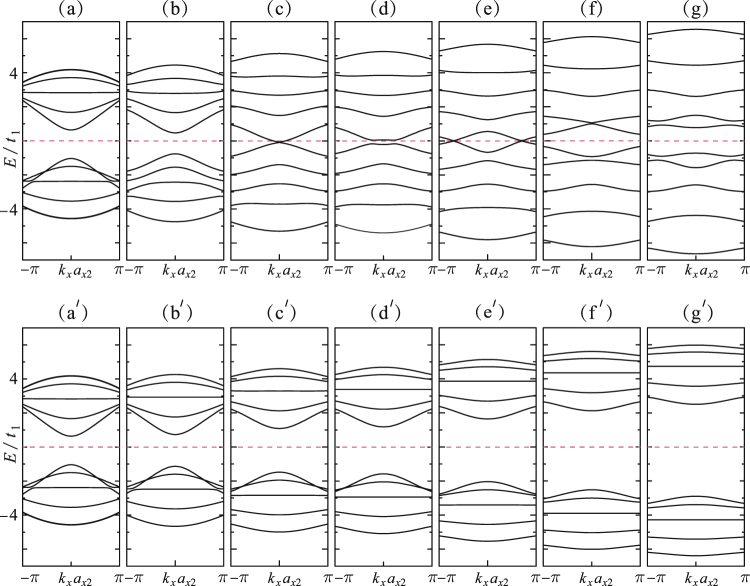
(**a**–**g**) Dispersion relations of 5a-Q1D-PNR as functions of the electric field *E*_*y*_. The relating electronic potential are respectively set for $4e{E}_{y}{a}_{0}\,\sin \,\frac{\alpha }{2}\,=\,0$, 1.5*t*_1_, 2.6*t*_1_, 4.0*t*_1_, 5.6*t*_1_, 7.0*t*_1_ and 8.0*t*_1_. (a′–g′) Dispersion relations as functions of *E*_*z*_ by setting the electronic potential *eE*_z_*a*_1_cos*β* = 0 1.5*t*_1_, 2.6*t*_1_, 4.0*t*_1_, 5.6*t*_1_, 7.0*t*_1_ and 8.0*t*_1_, respectively.

At last, we discuss the modulation of 5a-Q1D-PNR’s band gap by applying the external electric field. The cases of applying the *y*- and *z*-direction electric fields are respectively considered. The effect of applying the *y*-direction (along the width direction) electric field is to form the electronic potential difference between between the ribbon edges as ${V}_{y}\,=\,4e{E}_{y}{a}_{0}\,\sin \,\frac{\alpha }{2}$, while the *z*-direction field results in the potential difference between the two planes as *V*_z_ = *eE*_*z*_*a*_1_cos*β*. Modulations of the gap by applying electric fields also show strong direction dependence. The dispersion-relations for |*V*_*y*_| = 0, 1.5*t*_1_, 2.6*t*_1_, 4.0*t*_1_, 5.6*t*_1_, 7.0*t*_1_ and 8.0*t*_1_ are respectively shown in Fig. 12(a–g). The electronic response is nonlinear and turns out three variation regions with increasing |*V*_*y*_|: (i) Firstly, the band gap decreases with increasing the field strength [see Fig. 12(a,b)]. (ii) The band gap almost closes at about |*V*_*y*_| = 3.6*t*_1_ [see Fig. 12(c)], but interestingly, it opens again with further increasing |*V*_*y*_| [Fig. 12(d)] and closes again at |*V*_*y*_| = 5.6*t*_1_ [see Fig. 12(e)]. This variation was also previously predicted for 8a-Q1D-PNR by Sisakht^26^ and it was addressed that such a novel trend was a character of extremely narrow aPNRs. The last achieved metallic state exhibits two Dirac-like points. These two points are pushed toward *k* = ±*π* with increasing |*V*_*y*_|. (iii) After the two Dirac-like points reached *k* = ±*π*, further increasing *V*_*y*_ reversely increase the band gap [see Fig. 12(f,g)]. In contrast, when increasing the *y*-direction electric field strength [|*V*_*y*_| = 0, 1.5*t*_1_, 2.6*t*_1_, 4.0*t*_1_, 5.6*t*_1_, 7.0*t*_1_, 8.0*t*_1_ are respectively shown in Fig. 12(a′–g′)], the gap size monotonically keeps increasing.

## Discussions and Conclusions

In summary, based on the TB calculations, we comparatively studied the electronic and optical responses of 4z-Q1D-PNR and 5a-Q1D-PNR to strain and electric field. The results suggested that zigzag- and armchair-edge phosphorene nanoribbons had distinct response behavior and therefore they could be used as different functional devices.

Zigzag-edge Q1D-PNR exhibited the metallic ground state. The inter-plane strain played the central role to form adhesively paired bands near the Fermi level. Adjusting the magnitude of inter-plane strain would significantly influence the optical conductivity spectrum and induce the shift of the absorption peaks; on the contrary, the optical response to in-plane strain is relatively weak, but the bands’ dispersive character is sensitive to inter-plane strains so that the effective mass of electrons/holes could be significantly affected. On the other hand, for armchair-edge Q1D-PNR, which was an insulator with the direct band gap, we found that applying the compressive inter-plane strain would enlarge the gap, while the compressive in-plane strain would decrease the gap. No matter zigzag- or armchair-edge ones, it seemed that the topology of the electronic state was preserved against any strains, so to say, one should not expect a strain induced M-I or I-M transition in a Q1D-PNR.

To break the symmetry preserved topology of the electronic state, one could consider applying an electric field. We addressed that for zigzag-edge Q1D-PNR, applying the electric field either along the *y* or *z* direction would induce the M-I transition when the strength of the field reaches a threshold. Furthermore, it was showed that the key factor to determine the direct/indirect character of the field-induced gap was the e-h asymmetry, which could be controlled by adjusting the in-plane strains. On the contrary, for armchair-edge Q1D-PNR, we showed that both the *y*- and *z*-direction external electric field could modulate the gap size, but they behaved quite distinct modulating rules: increasing the *y*-direction electric field led to the non monotonic response of the gap with three variation regions, while increasing the *z*-direction electric field monotonically enlarged the gap. Our theoretical work provides a fundamental understanding of the electronic and optical properties of zigazag- and armchair-edge Q1D-PNRs in presence of strains and electric fields. We believe these results should be meaningful for engineering BP based quasi-one-dimensional molecular devices in the future.

## References

[1] Bridgman PW (1914). Two new modifications of phosphorus. J. Am. Chem. Soc..

[2] Li L (2014). Black phosphorus field-effect transistors. Nat. Nanotechnol..

[3] Liu H (2014). Phosphorene: an unexplored 2D semiconductor with a high hole mobility. ACS Nano.

[4] Reich ES (2014). Phosphorene excites materials scientists. Nature.

[5] Lu W (2014). Plasma-assisted fabrication of monolayer phosphorene and its Raman characterization. Nano Research.

[6] Chen X (2015). High-quality sandwiched black phosphorus heterostructure and its quantum oscillations. Nat. Commun..

[7] Koening SP, Doganov RA, Schmidt H, Castro Neto AH, Özyilmaz B (2014). Electric field effect in ultrathin black phosphorus. Appl. Phys. Lett..

[8] Xia F, Wamh H, Jia Y (2014). Rediscovering black phosphorus as an anisotropic layered material for optoelectronics and electronics. Nat. Commun..

[9] Qiao J, Kong XH, Hu ZX, Yang F, Ji W (2014). High-mobility transport anisotropy and linear dichroism in few-layer black phosphorus. Nat. Commun..

[10] Jain A, McGaughey AJH (2015). Strongly anisotropic in-plane thermal transport in single-layer black phosphorene. Sci. Rep..

[11] Qin G (2015). Anisotropic intrinsic lattice thermal conductivity of phosphorene from first principles. Phy. Chem. Chem. Phy..

[12] Buscema M (2014). Fast and broadband photoresponse of few-layer black phosphorus field-effect transistors. Nano Lett..

[13] Youngblood N, Chen C, Koester SJ, Li M (2015). Waveguide-integrated black phosphorus photodetector with high responsivity and low dark current. Nat. Photon..

[14] Guo Q (2016). Black phosphorus mid-infrared photodetectors with high gain. Nano. Lett..

[15] Rodin AS, Carvalho A, Castro Neto AH (2014). Strain-induced gap modification in black phosphorus. Phys. Rev. Letts..

[16] Peng X, Wei Q, Copple A (2014). Strain-engineered direct-indirect band gap transition and its mechanism in two-dimensional phosphorene. Phys. Rev. B.

[17] Quereda J (2016). Strong modulation of optical properties in black phosphorus through strain-engineered rippling. Nano Lett..

[18] Liu Q, Zhang X, Abdalla LB, Fazzio A, Zunger A (2015). Switching a normal insulator into a topological insulator via electric field with application to phosphorene. Nano Lett..

[19] Wang T (2015). Tunable bandgap of monolayer black phosphorus by using vertical electric field: A DFT study. J. Kore. Phys. Soc..

[20] Cao T, Li X, Liu L, Zhao J (2016). Electric field and strain tunable electronic structures in monolayer Black Phosphorus. Comp. Mater. Sci..

[21] Das PM (2016). Controlled sculpture of black phosphorus nanoribbons. ACS Nano.

[22] Xiao Z (2017). Deriving phosphorus atomic chains from few-layer black phosphorus. Nano Res..

[23] Rudenko AN, Katsnelson MI (2014). Quasiparticle band structure and tight-binding model for single-and bilayer black phosphorus. Phys. Rev. B.

[24] Rudenko AN, Yuan S, Katsnelson MI (2015). Toward a realistic description of multilayer black phosphorus: From GW approximation to large-scale tight-binding simulations. Phys. Rev. B.

[25] Ezawa M (2014). Topological origin of quasi-flat edge band in phosphorene. New J. Phys..

[26] Sisakht ET, Zare MH, Fazileh F (2015). Scaling laws of band gaps of phosphorene nanoribbons: A tight-binding calculation. Phys. Rev. B.

[27] Cakir D, Sahin H, Peeters FM (2014). Tuning of the electronic and optical properties of single-layer black phosphorus by strain. Phys. Rev. B.

[28] Peng X, Copple A, Wei Q (2014). Edge effects on the electronic properties of phosphorene nanoribbons. J. Appl. Phys..

[29] Guo H, Liu N, Dai J, Wu X, Zeng XC (2014). Phosphorene nanoribbons, phosphorus nanotubes, and van der Waals multilayers. J. Phys. Chem. C.

[30] Zhang LL, Yamamoto S (2014). Photoinduced directional and bidirectional phase transitions in bistable linear polycyclic aromatic compounds. J. Phys. Soc. Jpn..

[31] Hu T, Han Y, Dong J (2014). Mechanical and electronic properties of monolayer and bilayer phosphorene under uniaxial and isotropic strains. Nanotechnology.

[32] Kretinin A (2013). Quantum capacitance measurements of electron-hole asymmetry and next-nearest-neighbor hopping in graphene. Phys. Rev. B.

[33] Chung HC, Chang CP, Lin CY, Lin MF (2016). Electronic and optical properties of graphene nanoribbons in external fields. Phys. Chem. Chem. Phys..

[34] Ho YH, Tsai SJ, Lin MF, Su WP (2013). Unusual Landau levels in biased bilayer Bernal graphene. Phys. Rev. B.

[35] Lin YP, Wang J, Lu JM, Lin CY, Lin MF (2014). Energy spectra of ABC-stacked trilayer graphene in magnetic and electric fields. RSC Adv..

[36] Tran V, Yang L (2014). Scaling laws for the band gap and optical response of phosphorene nanoribbons. Phys. Rev. B.

[37] Kimouche A (2015). Electronic and optical properties of graphene nanoribbons in external fields. Nat. Commun..

[38] Son YW, Cohen ML, Louie SG (2006). Energy gaps in graphene nanoribbons. Phy. Rev. Lett..

[39] Tanaka K, Koike T, Ohzeki K, Yamabe T (1985). Electronic structures of polyacenacene and polyphenanthrophen anthrene. Design of one-dimensional graphite. Synth. Met..

[40] Wang X (2011). Superconductivity at 5 K in alkali-metal-doped phenanthrene. Nat. Commun..

[41] Shao DF, Lu WJ, Lv HY, Sun YP (2014). Electron-doped phosphorene: a potential monolayer superconductor *Europhys*. Letts..

[42] Ugeda MM, Brihuega I, Guinea F, Gomez-Rodriguez JM (2010). Missing atom as a source of carbon magnetism. Rhys. Rev. Letts..

[43] Sun K, Gu Z, Katsura H, Das Sarma S (2011). Nearly flatbands with nontrivial topology. Rhys. Rev. Letts..

[44] Nandkishore R, Levitov LS, Chubukov AV (2012). Chiral superconductivity from repulsive interactions in doped graphene. Nat. Phys..

[45] Lin MF, Shyu FL (2000). Optical properties of graphene nanoribbons. J. Phys. Soc. Jpn..

[46] Hsu H, Reichi LE (2000). Selection rule for the optical absorption of graphene nanoribbons. Phys. Rev. B.

[47] Chung HC, Lee MH, Chang CP, Lin MF (2011). Exploration of edge-dependent optical selection rules for graphene nanoribbons. Opt. Express.

